# 3D-Printed Bubble-Free Perfusion Cartridge System for Live-Cell Imaging

**DOI:** 10.3390/s20205779

**Published:** 2020-10-12

**Authors:** Daigo Terutsuki, Hidefumi Mitsuno, Ryohei Kanzaki

**Affiliations:** Research Center for Advanced Science and Technology, The University of Tokyo, 4-6-1 Komaba, Meguro-ku, Tokyo 153-8904, Japan; mitsuno@brain.imi.i.u-tokyo.ac.jp

**Keywords:** 3D-printing, millifluidics, live-cell imaging, perfusion, bioanalytical methods, odorant sensor

## Abstract

The advent of 3D-printing technologies has had a significant effect on the development of medical and biological devices. Perfusion chambers are widely used for live-cell imaging in cell biology research; however, air-bubble invasion is a pervasive problem in perfusion systems. Although 3D printing allows the rapid fabrication of millifluidic and microfluidic devices with high resolution, little has been reported on 3D-printed fluidic devices with bubble trapping systems. Herein, we present a 3D-printed millifluidic cartridge system with bent and flat tapered flow channels for preventing air-bubble invasion, irrespective of bubble volume and without the need for additional bubble-removing devices. This system realizes bubble-free perfusion with a user-friendly interface and no-time-penalty manufacturing processes. We demonstrated the bubble removal capability of the cartridge by continually introducing air bubbles with different volumes during the calcium imaging of Sf21 cells expressing insect odorant receptors. Calcium imaging was conducted using a low-magnification objective lens to show the versatility of the cartridge for wide-area observation. We verified that the cartridge could be used as a chemical reaction chamber by conducting protein staining experiments. Our cartridge system is advantageous for a wide range of cell-based bioassays and bioanalytical studies, and can be easily integrated into portable biosensors.

## 1. Introduction

The emergence of additive manufacturing techniques such as three-dimensional (3D) printing has had a significant impact on medical and biological research [1,2]. One key advantage of 3D printing in comparison with conventional manufacturing methods such as machining is that complex 3D structures can be built directly, with rapid prototyping and flexible design. These features mean that 3D printing has high potential to resolve various issues in biological research. The real-time monitoring of cellular activity via live-cell imaging in perfusion chambers holds a prominent position in modern cell biology research [3,4] and is applied to numerous biosensing techniques [5,6,7]. However, although live-cell imaging protocols regarding the experimental set-up and reagents are well established [4,8], air bubble accumulation is still a serious concern for perfusion chamber systems [9,10]. These bubbles can interrupt experimental procedures and nullify experimental results, but are difficult to remove from flow channels and observation areas [11]. Therefore, a method of removing or preventing bubble formation in fluidic systems is of considerable need.

In this study, we developed a bubble-free 3D-printed perfusion cartridge that enables cellular response measurements using an optical microscope. It has millimeter-scale bent and flat tapered flow channels that prevent air bubbles from entering the observation area without the need for additional bubble-removing devices. Various bubble trap systems based on microfluidic technologies have been proposed previously [11,12,13,14,15,16]. For instance, researchers have developed a two-layer bubble barrier structure, in which the top layer blocks bubbles and the bottom layer works as a fluidic channel [11], and a debubbler using a polydimethylsiloxane (PDMS) pneumatic layer [12]. Lochovsky et al. developed an in-plane bubble trapping strategy based on single-layer soft lithography [13]. However, these devices require additional vacuum systems or trapping components, which are particularly inconvenient for portable and integrated fluidic systems [17]. In addition, they require a time-consuming fabrication processes, such as the preparation of master molds and bonding of polydimethylsiloxane (PDMS) structures to glass via oxygen plasma treatment in a clean environment [18,19]. 3D printing has been used previously to develop microfluidic [20,21] and millifluidic [22,23] devices with improved usability [22,23,24]; however, none of these devices incorporated bubble-removing functionality. Nevertheless, the attributes of 3D printers make them suitable for the fabrication of devices with specialized fluidic structures that can prevent bubble invasion. Modern 3D printers have high *z*-axis resolutions [25], which facilitates the printing of hollow structures with dimensions of several hundred micrometers, and offers the possibility of forming complex 3D fluidic networks [26]. Straight flow channels can be produced by machining, albeit at relatively high cost [27], but complex fluidic networks can only be produced by 3D printing [28] or other more complex processing techniques. In addition, 3D-printed components can be fabricated from transparent materials to allow visualization of the flow in the fluidic channels. 

Live-cell imaging requires frequent cell sample replacements; therefore, microfluidic devices still require burdensome procedures such as sorting cells to the exact place for observation while maintaining appropriate concentrations and controlling the flow rate of the culture medium to limit cell adhesion [19]. Thus, we focused on developing a cartridge system with millifluidic channels (1–10 mm [22,23,24]) that could function as an easy-to-handle perfusion system for users who are not microfabrication experts. We assume that the cartridge could be applied for measurements that take several hours; however, long-term cell culture (e.g., overnight) measurements are outside the scope of this study.

To investigate the bubble-removing mechanisms of the cartridge, we first developed a transparent cartridge and visualized the flow in the fluidic channels. Next, we examined the bubble removal capabilities of the cartridge by periodic bubble insertion with different bubble volumes. We then conducted calcium imaging of genetically modified cells using the cartridge. Our group developed an odorant sensor element based on Sf21 insect cells expressing insect odorant receptors (ORs) with OR co-receptors (Orcos) and fluorescent calcium indicator proteins [5]. These cells were previously seeded in a commercial open bath chamber and their response was measured using fluorescence microscopy [5,6,7]. In these experiments, air bubbles were intentionally introduced between each odorant solution and assay buffer to prevent dilution of the odorant solution. 

For the development of portable odorant sensors, a closed perfusion chamber is desirable; however, no appropriate commercial perfusion chambers are available. Therefore, we applied the 3D-printed cartridge to these cells and compared the cellular responses with those identified using a commercial open bath chamber. We also demonstrated the wide-area measurement function of the 3D-printed cartridge by using a low-magnification objective lens. Cost-effective and easy-to-fabricate millifluidic devices have been used as platforms for organic, inorganic, and materials syntheses [22]. Thus, we finally conducted protein staining in the 3D-printed cartridge to demonstrate its potential as a chemical reactor. The results demonstrated that the 3D-printed cartridge with millifluidic channels had high bubble-removing ability and good operability. Moreover, the design philosophy can be applied to the development of a wide range of biological and chemical studies and biosensor systems.

## 2. Materials and Methods

### 2.1. Cartridge Design and Fabrication

3D computer-aided design (CAD) models of the cartridge were designed using Autodesk Inventor Professional 2019 software (Autodesk, Inc., San Rafael, CA, USA). The CAD data were exported in STL format for the 3D printer. A ProJet MJP 3600 MAX 3D printer (3D Systems, Rock Hill, SC, USA) in its maximum resolution mode (XHD mode; *z*-axis resolution of 16 μm) was used for cartridge printing using a translucent and biocompatible ultraviolet (UV)-curable acrylic resin (Visijet M3 Crystal; 3D Systems). The cartridge printing process took approximately 4 h in the XHD mode. The cartridge is composed of separate top and bottom parts. We also fabricated a transparent 3D-printed cartridge using the same CAD data for visualizing flow in the channels. Owing to material (RGD−810 VeroClear; Stratasys, Eden Prairie, MN, USA) and printer (Objet500 Connex3; Stratasys) limitations, transparent materials were printed with a 30 µm *z*-axis resolution.

### 2.2. Computational Fluid Dynamics (CFD) Simulation

Flow dynamics in the designed cartridge were simulated using commercial computational fluid dynamics (CFD) software (ANSYS 18; ANSYS, Inc., Canonsburg, PA, USA). After importing the CAD model of the cartridge into ANSYS, a tetrahedral mesh was applied to the fluid region. The mesh size was 200 µm and the fluid boundary region was composed of a 20-layer mesh. The assay buffer passing through the cartridge was approximated as water and the mass flow rate of the inlet was set to 2.5 × 10^−5^ kg/s.

### 2.3. Cells and Odorants

We used Or13a cells, which are Sf21 cells derived from the pupal ovarian tissues of *Spodoptera frugiperda* that express an OR from *Drosophila melanogaster* that strongly responds to 1-octen-3-ol with Orcos and fluorescent calcium indicator proteins (GCaMP6s). The cells were cultured in Grace’s insect medium (Invitrogen, Carlsbad, CA, USA) at 27 °C. The odorant 1-octen-3-ol (>98% purity; O5284-25G; Sigma-Aldrich Co., St. Louis, MO, USA) was diluted with assay buffer solution (140 mM NaCl, 5.6 mM KCl, 4.5 mM CaCl_2_, 11.26 mM MgCl_2_, 11.32 mM MgSO_4_, 9.4 mM D-glucose, and 5 mM HEPES; pH 7.2) containing 0.1% dimethyl sulfoxide (DMSO; Wako Pure Chemical Industries, Ltd., Osaka, Japan) for odorant stimulations. The Sf21 cell line derived from *S. frugiperda* was purchased from Life Technologies (Life Technologies, Carlsbad, CA, USA). All genetic experiments in this research were followed life science research ethics and safety of the University of Tokyo.

### 2.4. Device Assembly and Cell Seeding

A circular cover glass (diameter: 22 mm; thickness: 0.13–0.17 mm; Matsunami, Osaka, Japan) was inserted in the top observation area and fixed by a thick O-ring. In order to prevent assay buffer leakage, a small O-ring was installed between the circular groove of the top component and the circular cover glass. A square cover glass (dimensions: 18 × 18 mm^2^; thickness: 0.13–0.17 mm; Matsunami) was installed in the fixed area of the bottom component, and a small O-ring was inserted into the circular groove of the bottom component. Finally, four M3 acrylic screws securely joined the top and bottom of the cartridge. After assembling the cartridge, Or13a cells in 150-µL suspensions were manually seeded on the square cover glass in the designed cartridge using a micropipette and incubated at room temperature (approximately 23–25 °C) for 1 h. Sf21 cells can be cultured at a wide room temperature range (19–28 °C) without CO_2_ [29], so the cartridge and cells were simply stored on a desk.

### 2.5. Calcium Imaging

The activity of Or13a cells seeded on the cover glass in the cartridge was evaluated by calcium imaging in the presence of odorants at different concentrations. Metal tubes (PT-QE1; Warner Instruments, Hamden, CT, USA) held by tube clamps (CAT-1; Narishige Co. Ltd., Tokyo, Japan) were connected to the inlet and outlet of the 3D-printed cartridge. Silicone tubes with an inner diameter of 1 mm were connected to the metal tubes and the inlet side of a peristaltic pump (MP-2000B; EYELA, Tokyo, Japan), which circulated the assay buffer solution with 0.1% DMSO at a flow rate of approximately 1.5 mL/min. The flow rate of the outlet side of the peristaltic pump was set slightly higher than 1.5 mL/min (2.0–2.5 mL/min). Each odorant was applied for 15 s with the assay buffer solution and 0.1% DMSO. To record the odorant responses of the Or13a cells via fluorescence signals, an optical microscope (BX51WI; Olympus, Tokyo, Japan) with an objective lens with a long working distance (WD) (LUCPLFLN 20×; WD: 6.60–7.80 mm; Numerical Aperture: 0.45; Olympus), a fluorescence mirror unit for green fluorescent protein (GFP) (U-MGFPHQ; Olympus), and a charge-coupled device camera (DU-897E; Andor Technology PLC, Belfast, UK) were used. Or13a cells in the 3D-printed cartridge were selected in the region of interest (ROI) based on the circular Hough transform [30,31] using MATLAB (MathWorks, Natick, MA, USA) after capturing video images from the fluorescence measurements. The fluorescence intensity change Δ*F*/*F*_0_ was defined as

(1) $$\frac{\Delta F}{F_{0}}\;\left[\%\right]=\;\frac{F_{t}\;-\;F_{0}}{F_{0}}\times 100\;$$

where *F_t_* is the fluorescence intensity at time *t* and *F*_0_ is the fluorescence intensity measured before odorant stimulation. The average fluorescence intensity change was defined as subtracting a 20 s average of the baseline before reaching odorant stimulation from a 20 s average of the peak value.

Calcium imaging was also conducted using a commercial open bath chamber with the following conditions. The Or13a cells were seeded on a 12 mm diameter coverslip and cultured at room temperature for approximately 1 h. Subsequently, the coverslip with Or13a cells was inserted into a quick-change chamber (RC-48LP; Warner Instruments). To record the response of the Or13a cells, a water-immersion objective lens (UMPLFLN 20×; WD: 3.5 mm; NA: 0.5; Olympus) was inserted into the assay buffer in the chamber. The other experimental and analysis conditions were the same as in the 3D-printed cartridge experiment.

## 3. Results

### 3.1. Design and Operation of the 3D-Printed Perfusion Cartridges

**Figure 1:**
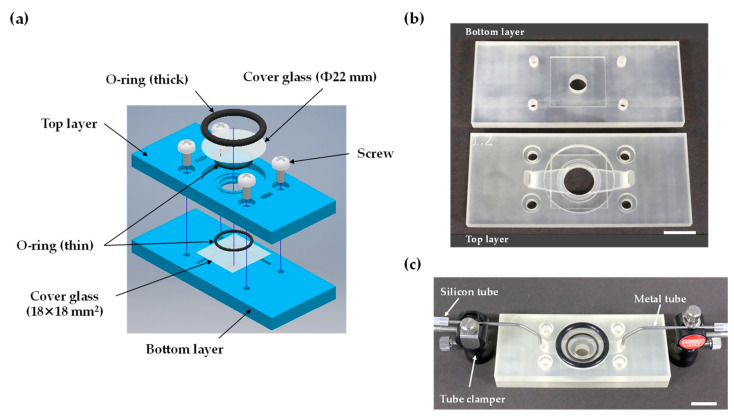
Design and setup of the 3D-printed perfusion cartridge system: (**a**) assembly view of the 3D-printed cartridge; (**b**) two-layer 3D-printed cartridge parts. The top layer includes flow channels and the observation area. The bottom part works as a base; (**c**) cartridge setup for assay buffer perfusion with metal tubes connected to silicone tubes and fixed by tube clampers. All scale bars are 10 mm.

The 3D-printed cartridge was designed to reproduce the capabilities and functions of previous calcium imaging experiments on insect cells expressing insect ORs using commercial open bath chambers [5,6,7]. The 3D-printed cartridge was built as two separate layers. The top layer was 30 mm long, 70 mm wide, and 5.5 mm thick, and comprised the cell culture area, observation window for imaging, perfusion flow channels, four through holes, and a circular groove for fixing a thick O-ring. The cell culture volume for observation was 236 µL (diameter: 10 mm; height: 3 mm) and the flow channel in the horizontal direction enclosed approximately 90 µL of assay buffer. The perfused assay buffer volume using the open bath chamber was adjusted to approximately 300 µL during calcium imaging, and the 3D-printed cartridge could perfuse nearly the same volume of assay buffer. The bottom layer of the 3D-printed cartridge was 30 mm long, 70 mm wide, and 5 mm thick, and comprised a recessed area for an 18 × 18 mm^2^ square cover glass to seed cells, four screw holes, and a circular groove for fixing a thin O-ring. The CAD assembly drawing of the cartridge is shown in Figure 1a. The 3D-printed cartridge parts and device setup with tubes are shown in Figure 1b,c. Two metal tubes held by tube clampers are connected to the inlet and outlet holes of the cartridge for assay buffer perfusion (Figure 1c).

Most existing commercial closed chambers are equipped with narrow stainless-steel tubes having simple circular cross-sections. They have little or no bubble removal capability. Previous microfluidic devices capable of removing bubbles were equipped with complex trapping structures or membrane filters. The 3D-printed cartridge developed in this study simply utilizes the buoyancy of the bubbles and is equipped with bent and flat tapered flow channels for preventing bubble invasion. The vertical portion of the bent channel for perfusion is a pathway for bubbles moving against gravity and rising to the surface of the inlet. The horizontal portion of the bent channel structurally prevents bubble invasion from the vertical direction. To simply realize these structures for bubble removal, we used 3D printing technology that offers the possibility of forming 3D fluidic networks. The details of the flow channels in the 3D-printed cartridge are provided below. The flow channels have a 70° bend in the longitudinal direction and their cross-section is a rounded rectangle with a cross-sectional area of approximately 10 mm^2^. The horizontal section of the flow channel also has a rounded rectangle cross-section and an inverted taper toward the observation area. The inverted taper structure enables the flow rate of the inlet hole of the observation area to be the same as the flow rate of the inlet hole of the cartridge surface, and helps liquids such as the assay buffer and odorant stimuli to spread evenly in the observation area. The cross-sectional area of the inlet hole on the top part is approximately 10 mm^2^. These structures allow both efficient bubble removal and high flow rates. Cross-sectional views of the 3D-printed cartridge are shown in Figure S1, Supplementary Materials. 

3D printing provides excellent unibody construction. However, multipart design can enhance the user-friendliness of 3D-printed objects [32]. The cartridge in this study employed a two-part design that is held together by four screws. Separate components allow cells to be easily seeded, easy replacement of the components after experiments, and reduced risk of leakage. When the flow channels or observation area structures need to be modified, only the top part needs to be replaced; the bottom part can be reused. After an experiment, a commercial neutral detergent (e.g., Contaminon; FUJIFILM Wako Pure Chemical Corporation, Osaka, Japan) can be used to wash the flow channels and surfaces of the cartridge. Although most microfluidic devices are not reusable [19], the 3D-printed cartridge had excellent reusability, and withstood washing dozens of times.

### 3.2. Bubble Removal Ability of the 3D-Printed Perfusion Cartridge

**Figure 2:**
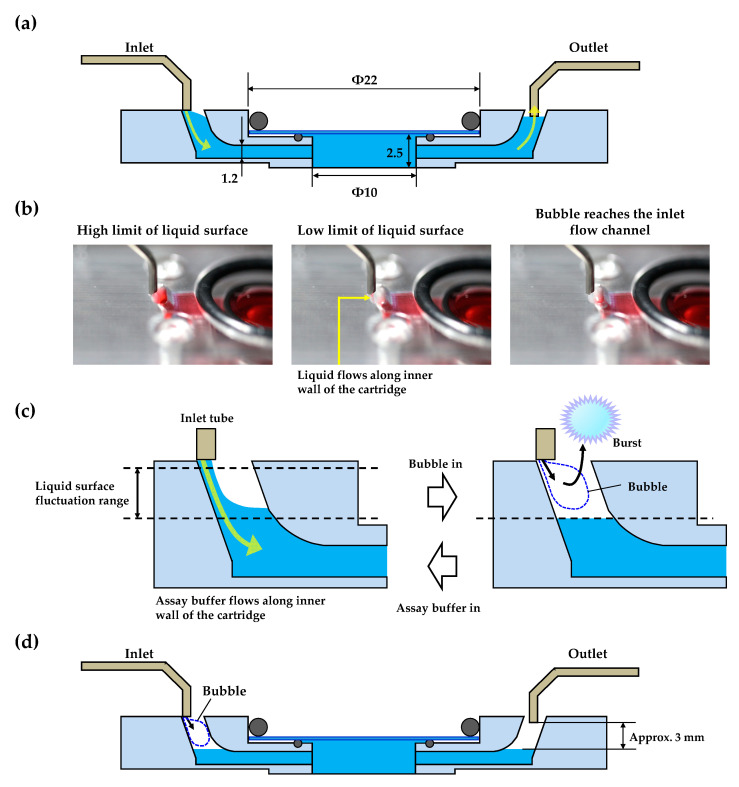
(**a**) Schematic of cross-sectional view of the assembled top-layer of the 3D-printed cartridge with metal tubes during perfusion; (**b**) images of the liquid surface level; (**c**) schematic of the bubble removal mechanism of the top-layer of the 3D-printed cartridge; (**d**) schematic of medium retention mechanism of the 3D-printed cartridge.

The most important feature of the designed 3D-printed perfusion cartridge is its efficient removal of air bubbles due to the wide 3D-printed flow channels. Most existing commercial closed chambers for live-cell imaging have little or no bubble removal ability and require some sort of pre-treatment to prevent bubbles. We designed the 3D-printed perfusion cartridge with bent and flat flow channels to prevent air bubble formation. Our focus was to remove the visible bubbles that originate from outside the cartridge. A schematic diagram of the cross-section of the top side designed cartridge with metal tubes is shown in Figure 2a. The inlet-side metal tube tip is in light contact with the inlet hole of the cartridge, while the tip of the metal tube on the outlet side is inserted 0.5–1 mm into the outlet hole. 

To clarify the mechanism of bubble removal from the cartridge, we fabricated a transparent cartridge and perfused ultra-pure water colored with red food dye. Images of the liquid surface levels are shown in Figure 2b, and a video clip is available in the Supplementary Materials (Video 1). This test showed that there were maximum and minimum fluctuation levels for the liquid surface in the longitudinal direction of the flow channel. An enlarged schematic diagram of the inlet side of Figure 2a is shown in Figure 2c to describe the mechanism of bubble removal. During perfusion, the liquid surface on the inlet side fluctuated between the maximum and minimum levels. In this case, bubbles from the inlet tube do not reach the observation area because they cannot pass through the horizontally directed flow channels. The bubbles therefore disintegrate in the vertically directed flow channels or outside the cartridge. The liquid surface on the outlet side is automatically drawn toward the outlet-side flow channel; however, this surface did not exceed the height of the outlet-side metal tube tip because the flow rate of the outlet peristaltic pump was set to greater than 1.5 mL/min (approximately, 2.0–2.5 mL/min). There is a gap (approximately 3 mm) between the horizontal level of the medium and the tip of the outlet-side metal tube (Figure 2d) in the 3D-printed cartridge. Therefore, the outlet pump does not draw the medium from the observation area or the horizontal direction flow channel when large bubbles are introduced from the inlet side, or even if perfusion is stopped. This is one of the advantages of our system. 

To investigate the volume of the bubbles that the cartridge can remove, we intentionally generated air bubbles and introduced them into the cartridge for 1 min at ambient temperature. The bubble volume was adjusted by changing the speed at which the silicone tube was pulled out of and reinserted into the ultrapure water with a pump flow rate of 1.5 mL/min. Small bubbles (< 1 µL) were generated uniformly and continuously using a commercial aquarium air stone, and over 100 bubbles were introduced into the 3D-printed cartridge. Table 1 presents the bubble volume information and bubble removal results. Video 2 (Supplementary Materials) shows that the cartridge prevents air bubbles from entering the observation area. We also measured the maximum flow rate that the cartridge can support; the results show that the cartridge prevented air bubbles up to a fluid flow rate of 4 mL/min. Although perfusion flow rates depend on the size of the chambers or the objectives and type of experiment, a perfusion flow rate up to 3 mL/min is generally sufficient for calcium imaging using commercial chambers to rapidly exchange buffer solution without disturbing cells attached on a coverslip [8]. We used commercial tube clamps to fix the metal tubes and adjust their tip positions; however, the 3D-printed fixtures (Figure S2, Supplementary Materials) facilitate the positioning of the metal tube tips. Given that the cartridge developed in this study can remove a wide range of bubble volumes without any additional devices, it is well suited to live-cell imaging for biological research and portable biosensor systems.

In various biological assays, DMSO is a standard organic solvent for dissolving neuroscience research reagents [33] or odorant molecules [5,6,7]. Thus, the 3D-printed cartridge must be compatible with organic solvents. DMSO concentrations in the range of 0.5–1.5% are widely used [34], whereas DMSO concentrations of 5% and greater significantly inhibit cell viability [33] and are not appropriate for cell-based bioassays. We selected 5% and 10% DMSO concentrations in the assay buffer for bubble prevention tests under the same set-up as that shown in Supplementary Materials Video 2. The 3D-printed cartridge prevented all air bubbles from invading the observation area, despite the high concentration of organic solvents. Therefore, organic solvents can be used with the cartridge without leakage because the mechanism for bubble removal is based on the flow channel structure. These advantages are useful for both fundamental laboratory research and for developing practical and portable cell culture/imaging biosensors. 

For biosensor applications, our 3D-printed cartridge can potentially enable downsizing of sensor systems. Previous calcium imaging methods for insect cells expressing insect ORs required a large fluorescence microscope and a commercial open bath chamber that was difficult to integrate into sensor systems. In this study, we developed a 3D-printed closed perfusion chamber with high bubble removal ability. We expect to integrate it with a microperfusion pump in a portable fluorescence microscope or a photomultiplier tube module. These configurations will reduce the weight and size of sensor systems. This 3D-printed cartridge design can also be applied in perfusion systems of not only imaging biosensors but also electrical-signal-based biosensors [35,36]. Moreover, we performed odorant molecule detection and chemical reactions using the 3D-printed cartridge and the results are discussed in the following section; therefore, the cartridge could contribute toward developing portable odorant biosensing systems.

### 3.3. Calcium Imaging of Or13a Cells using the Designed Cartridge

**Figure 3:**
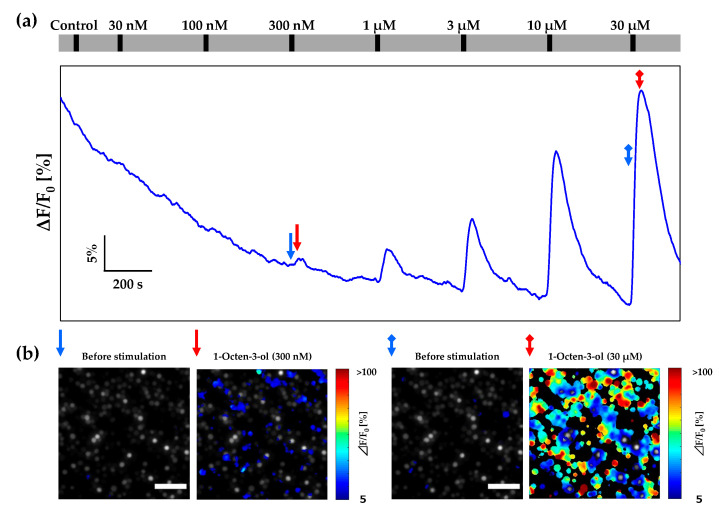
(**a**) Typical dose–response profiles of fluorescence intensity changes of Or13a cells in the 3D-printed cartridge during addition of 1-octen-3-ol at concentrations ranging from 30 nM to 30 µM. These responses indicated average numbers of Or13a cells in the fluorescence-microscope field of view. The long grey bar indicates the perfusion of the assay buffer solution including 0.1% DMSO by the peristaltic pumps. The short black bars indicate when each odorant flowed inside the chamber for 15 s, and assay buffer solution including 0.1% DMSO was used as a control. (**b**) Pseudo-color heat maps of cell-response rates in the 3D-printed cartridge exhibited a > 5% increase in fluorescence intensity before and after stimulation with 300 nM and 30 µM 1-octen-3-ol. The blue arrow and the blue arrow with a square tail in (**a**) indicate fluorescence intensity before 300 nM and 30 µM 1-octen-3-ol stimulation, respectively, and correspond to the same arrows of the heat map shown in (**b**). The red arrow and the red arrow with a square tail in (**a**) indicate fluorescence intensity change peaks induced by stimulation with 300 nM and 30 µM 1-octen-3-ol, respectively, and correspond to the same arrows of the heat map shown in (**b**). All scale bars are 100 µm.

In the previous section, we confirmed the bubble removal capabilities of the 3D-printed cartridge. To apply this system to live-cell imaging, we measured the fluorescence intensity changes of Sf21 cells expressing insect ORs using the cartridge. The dose response of Or13a cells toward 1-octen-3-ol doses ranging from 30 nM to 30 μM using the designed cartridge was investigated with a 20× objective lens. A typical dose–response profile for Or13a cells using the cartridge is shown in Figure 3a. Pseudo-color heat maps before and after 300 nM and 30 µM 1-octen-3-ol stimulations are shown in Figure 3b. From Figure 3a, it can be observed that the dose–response profile of Or13a cells was not affected by noise or distortions caused by bubble invasions, and fluorescence intensity changes were clearly detected. The total measurement time was 45.5 min with eight stimulations including intentionally introduced air bubbles. These results indicate that the designed cartridge is stable against periodic bubble invasions and is reliable over long operation times. 

We were concerned that a closed cartridge could reduce the sensitivity of Or13a cells. However, these results indicated that the dose-dependent responses of Or13a cells were consistent with those from previous studies. While the Or13a cells did not respond to 30 nM and 100 nM 1-octen-3-ol stimulations, the clear-cut response of Or13a cells toward the 300 nM odorant stimulation was detected in the response profile and the Or13a cells exhibited fluorescence intensity changes of more than 5% after stimulation, as shown in Figure 3b.

### 3.4. Calcium Imaging of Or13a Cells using a Commercial Open Chamber

**Figure 4:**
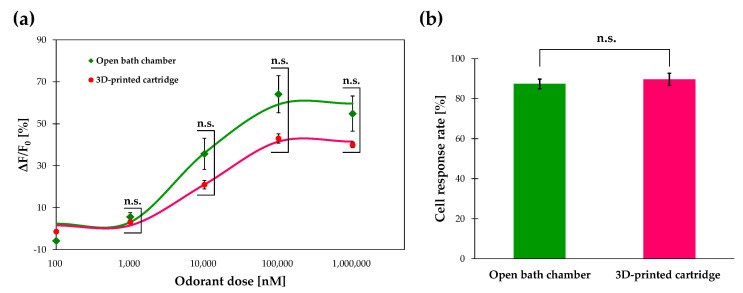
(**a**) Comparison between average dose-dependent response of Or13a cells in the 3D-printed cartridge with a 20× objective lens and in a commercial open bath camber with a 20× water-immersion objective lens previously used for Sf21 cells expressing insect ORs. The data represent the mean ± standard error of the mean (SEM) of the fluorescence intensity changes in all cells (*N* = 4 individual tests, Welch’s *t*-test; n.s. = not significant). (**b**) Comparisons of the cell-response rates in the 3D-printed cartridge and a commercial open bath chamber exhibited greater than 5% increase in fluorescence intensity during 10 μM 1-octen-3-ol stimulation. Data represent the mean ± SEM of fluorescence intensity changes of Or13a cells (*N* = 4 individual tests, Welch’s *t*-test: n.s., not significant).

To qualitatively assess the performance of the 3D-printed cartridge, we conducted calcium imaging of the Or13a cells using a commercial open bath chamber (RC-48LP; Warner Instruments) with the same 20× water-immersion objective lens as that used in previous studies [5,6,7] as a benchmark. The dose responses of Or13a cells toward 1-octen-3-ol doses ranging from 100 nM to 1 mM in the cartridge and a commercial open bath chamber were compared (Figure 4a). The dose–response curve of Or13a cells in the 3D-printed cartridge and the commercial open bath chamber exhibited EC50 values of 10.06 μM and 8.37 μM with average maximum responses of (42.89 ± 2.21)% and (64.11 ± 8.81)%, respectively. In this comparison, the detection limits of Or13a cells in both devices were the same as that of a 1 μM 1-octen-3-ol stimulation that derived a significantly different response from the control stimulation with a Welch’s *t*-test value of *p* < 0.05. Next, the cell-response rates of the Or13a cells in the 3D-printed cartridge with a 20× objective lens were compared with those using the commercial open bath chamber. More than 80% of Or13a cells in the 3D-printed cartridge exhibited a greater than 5% increase in fluorescence intensity when stimulated with 10 μM 1-octen-3-ol; the response rates were nearly the same as those in a commercial open bath chamber (Figure 4b). Hence, we demonstrated that the 3D-printed cartridge could be used in the same way as a commercial open bath chamber for the calcium imaging of Sf21 cells expressing insect ORs without affecting the detection sensitivity of the cells. The successful development of a closed cartridge with a non-water immersion objective lens is expected to expand cell measurement methods and lead to the development of portable biosensing systems.

### 3.5. Response Time and Recovery Time

For measurements of cell response, the fluorescence intensity of cells must be returned to baseline values. We evaluated the response and recovery times of fluorescence intensity changes in Or13a cells using the cartridge and a commercial open bath chamber. According to the definition of sensor parameters [37], the response time was defined as the time required for the fluorescence intensity of Or13a cells to increase from 10% to 90% of the maximum value during the addition of the odorant for 15 s at a flow rate of 1.5 mL/min. The recovery time was likewise defined as the time required for the fluorescence intensity of these cells to decrease from 90% to 10% of the maximum value. The response baselines were defined as the average fluorescence intensity measured during the 20 s before odorant stimulation. 

**Figure 5:**
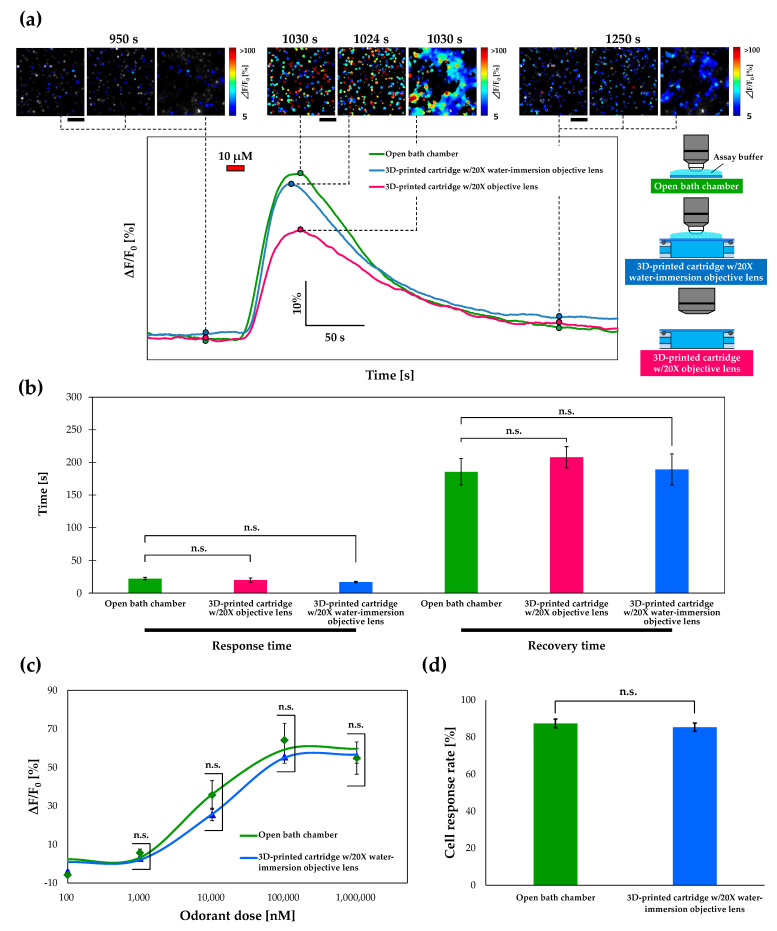
(**a**) Typical fluorescent response of the Or13a cells using the 3D-printed cartridge and a commercial open bath chamber when 10 µM 1-octen-3-ol stimulations were applied. Upper pseudo-color heat maps of cell-response rates (before stimulation, the peak of fluorescent intensity change, and after stimulation) in a commercial open bath chamber and the 3D-printed cartridge with a 20× water-immersion objective lens or a 20× objective lens exhibited a > 5% increase in fluorescence intensity from the baseline. We defined the start time as 0 s. (**b**) Comparisons between the average response and recovery times for Or13a cells in a commercial open bath camber and in the 3D-printed cartridge with a 20× water-immersion objective lens or a 20× objective lens. Data represent the mean ± SEM of fluorescence intensity changes in all cells (*N* = 3 individual tests, Welch’s *t*-test: n.s. = not significant). (**c**) Comparison between the average dose-dependent responses of Or13a cells in the 3D-printed cartridge with a 20× water-immersion objective lens and in a commercial open bath chamber. The data represent the mean ± SEM of the fluorescence intensity changes in all cells (*N* = 4 individual tests, Welch’s *t*-test; n.s. = not significant). (**d**) Comparison of the cell-response rates of the cells in the 3D-printed cartridge with a 20× water-immersion objective lens and those in a commercial open bath chamber. The cells in the 3D-printed cartridge exhibited a greater than 5% increase in fluorescence intensity during 10 μM 1-octen-3-ol stimulation. Data represent the mean ± SEM of the fluorescence intensity changes of the Or13a cells (*N* = 4 individual tests, Welch’s *t*-test: n.s., not significant).

Typical fluorescence intensity changes, response times, and recovery times with pseudo-color heat maps are shown in Figure 5a for the commercial open bath chamber and the 3D-printed cartridge with a 20× water-immersion objective lens or 20× objective lens. The average response time of Or13a cells stimulated by 10 µM 1-octen-3-ol using an open bath chamber was 22.3 ± 1.7 s and the average response times using the 3D-printed cartridge with the 20× objective lens and 20× water-immersion objective lens were 20.0 ± 3.2 s and 17.0 ± 1.0 s, respectively (Figure 5b). The average recovery time of these cells using an open chamber was 185.7 ± 20.3 s, while the average recovery times using the cartridge with the 20× objective lens and 20× water-immersion objective lens were 208.0 ± 16.2 s and 189.3 ± 23.7 s, respectively (Figure 5b). These results indicate that both the response and recovery times of fluorescence intensity changes of Or13a cells using the designed cartridge were nearly the same as those obtained using the commercial open bath chamber.

In the case of the 3D-printed cartridge with the 20× objective lens, halation in the pseudo-color heat maps of the Or13a cells and a decrease in the intensity change of the fluorescence peak occurred compared with the experiments using the open bath chamber (Figure 5a). These phenomena can also be observed in Figure 3b. Reflective excitation of Or13a cells by the 22 mm diameter cover glass and the difference in refractive indices between the assay buffer and air are cited as possible causes. Thus, we deposited an assay buffer on the 22 mm diameter cover glass of the 3D-printed cartridge and conducted calcium imaging using the 20× water-immersion objective lens to match the refractive indices. Consequently, the pseudo-color heat maps of the open bath chamber and the 3D-printed cartridge with the 20× water-immersion objective lens were similar (Figure 5a). Average dose-dependent response comparisons between the cartridge with the 20× water-immersion objective lens and open bath chamber of Or13a toward 1-octen-3-ol doses ranging from 100 nM to 1 mM were not statistically significant (Figure 5c). In addition, the mean values for the cartridge with the 20× water-immersion objective lens were closer to those for the open bath chamber compared with the results presented in Figure 4a (see Figure 5c). We again compared the cell-response rates of Or13a cells in the 3D-printed cartridge with the 20× water-immersion objective lens with those of the cells in the commercial open bath chamber, similar to the case shown in Figure 4b (Figure 5d). More than 80% of Or13a cells in the 3D-printed cartridge with the 20× water-immersion objective lens exhibited a >5% increase in fluorescence intensity when stimulated with 10 μM 1-octen-3-ol, and the response rate was not statistically significant compared with that of the cells in the commercial open bath chamber. 

We subsequently observed that the outline of cells in the fluorescence images taken from the 3D-printed cartridge with the 20× objective lens was blurred compared with those taken using the 20× water-immersion objective lens. We therefore applied the “Plot Profile” command with a one-pixel profile line width in ImageJ software (http://imagej.nih.gov/ij/) to the enlarged original fluorescence images measured using the two lenses. In the case of the measurement carried out using the 20× water-immersion objective lens, the brightness value of the black background was almost zero; in contrast, the black background for the measurement carried out using the 20× objective lens exhibited a brightness value of 10–20 (Figure S3, Supplementary Materials). These results indicate that halation can be reduced and that the intensity change of the fluorescence peak can be recovered by using the 20× water-immersion objective lens while retaining at least 80% of the cell response rate. When the 20× objective lens was used, each cell in the bright field image could be precisely tracked using MATLAB and the response profile of the Or13a cells did not include noise. Therefore, the 3D-printed cartridge could be used for calcium imaging without being affected by halation. In this section, we developed the 3D-printed cartridge with 0.8 mm deep flow channels to conduct calcium imaging because of the limitation in the focal length of the 20× water-immersion objective lens.

### 3.6. Wide-Area Observation

**Figure 6:**
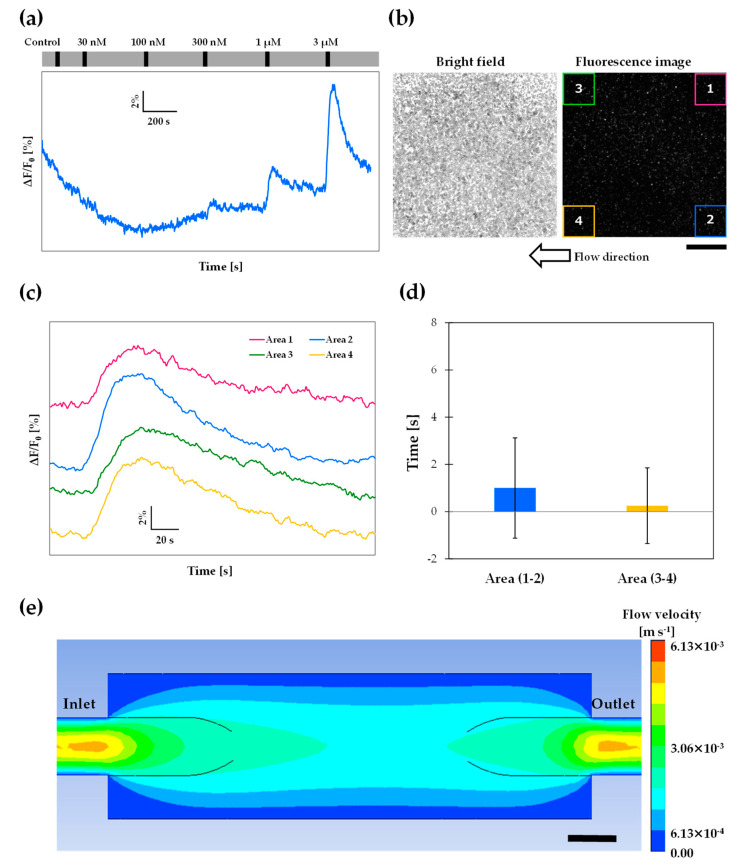
(**a**) Typical dose–response profiles of fluorescence intensity changes in Or13a cells in the 3D-printed cartridge using a 4× objective lens during the addition of 1-octen-3-ol at concentrations ranging from 30 nM to 3 µM. The long grey bar indicates the perfusion of the assay buffer solution including 0.1% DMSO by peristaltic pumps. (**b**) Bright field and baseline fluorescence images of Or13a cells observed by a 4× objective lens. (**c**) Each point of fluorescence intensity change in (**b**) observed by a 4× objective lens. The size of each observation field is 400 × 400 µm^2^. (**d**) Difference between response times of Area 1 and Area 2, and differences between response times of Area 3 and Area 4 stimulated by 3 µM 1-octen-3-ol. Data represent the mean ± SEM of calculated time differences between response time of each 400 × 400 µm^2^ area (*N* = 4 individual tests). (**e**) CFD simulation result of flow velocities in the 3D-printed cartridge. These velocities of the cross-sectional view of B–B direction in Figure S1, Supplementary Materials. (**e**) is represented as a contour. Scale bars: (**b**) 500 µm; (**e**) 1 mm.

One of the advantages of a closed cartridge is that the surface of the observation area is kept flat by a glass or plastic cover. This feature is useful for wide-area observation because a non-aqueous-immersion low-magnification objective lens can be used. Taking advantage of the designed cartridge, we conducted fluorescence measurements of Or13a cells using a non-aqueous-immersion low-magnification objective lens (UPLANFLN 4×; WD: 17 mm; NA: 0.13; Olympus). Typical dose–response profiles of fluorescence intensity changes measured using the 4× objective lens are shown in Figure 6a. With this low-magnification objective lens, cell detection using MATLAB did not work because the cells were too small. Therefore, we directly applied the “Plot Z-axis Profile” command in ImageJ to the observation field (2 × 2 mm^2^). The response profile indicated that the response of Or13a cells toward 300 nM odorant stimulation was clearly detectable. 

We next visualized the multipoint fluorescence intensity changes in the observation field using the 4× objective lens to characterize the fluid flow in the observation area. Bright field and baseline fluorescence images of Or13a cells in the 3D-printed cartridge observed by the 4× objective lens are shown in Figure 6b, in which the flow direction was right to left. Typical fluorescence intensity changes of Or13a cells stimulated by 3 µM 1-octen-3-ol at 4 points in a 400 × 400 µm^2^ area are shown in Figure 6c. If the assay buffer in the observation area were to flow in a parallel direction, there would be a time difference in the odorant responses between the upstream and downstream stimulations. To evaluate these differences, we calculated the response time during the addition of the odorant for 15 s at a flow rate of 1.5 mL/min. To estimate the flow speed of the assay buffer in the observation area, we calculated the time difference between the response time of Areas 3 and 1 and the time difference between the response time of Areas 4 and 2, which were 3.25 ± 1.31 s and 4.00 ± 1.08 s, respectively. Using these time differences, the flow speed between Areas 1 and 3 (distance: 1600 µm) was calculated to be 4.92 × 10^−4^ m/s and the flow speed between Areas 2 and 4 was calculated to be 4.00 × 10^−4^ m/s. We also calculated the time difference between the response times of Areas 1 and 2 and the time difference between the response times of Areas 3 and 4, which were 1.00 ± 2.12 s and 0.25 ± 1.60 s, respectively (Figure 6d). These results verified that the assay buffer and odorant in the observation area both flowed in parallel between the line connecting Areas 1 and 2, and the line connecting Areas 3 and 4. The fluid flow in the 3D-printed cartridge behaved similarly as that in an open bath chamber.

CFD simulation estimated the flow velocity in the designed cartridge, which was then compared with experimental data. When the mass flow rate of the inlet was 2.5 × 10^−5^ kg/s (equal to 1.5 mL/min), the flow velocity between the bottom of the observation area and approximately 300 µm above was 6.13 × 10^−4^ m/s (Figure 6e). The simulated velocity (6.13 × 10^−4^ m/s) was approximately equal to the experimental velocity (4.00 × 10^−4^ m/s), and the Or13a cells had this flow velocity because the heights of the Sf21 cells attached to the flat surface were approximately 10 µm [7]. These results suggest that CFD simulation could aid in designing 3D-printed cartridges.

### 3.7. 3D-Printed Cartridge as a Chemical Reactor

3D-printed millifluidic and microfluidic systems using PDMS have excellent potential for applications in chemical reaction and synthesis [22,38]. As an example of chemical reaction, we conducted protein staining using our cartridge system. Blocking agent (50 mg; ECL Prime blocking agent; GE Healthcare, Chicago, IL, USA) was dissolved in 1 mL PBS (D-PBS (-); FUJIFILM Wako Pure Chemical Corporation, Osaka, Japan) and three 2 µL droplets were deposited on the 18 × 18 mm^2^ cover glass. To identify the position of protein spots, four black dots were drawn on the back side of the cover glass. After setting the cover glass in the 3D-printed cartridge (as in the calcium imaging set-up), the cartridge was filled with assay buffer solution. Then, Coomassie blue dye (Bio-Safe Coomassie G-250 Stain; Bio-Rad Laboratories, Hercules, CA, USA) was circulated at a flow rate of approximately 0.2 mL/min for 10 min.

**Figure 7:**
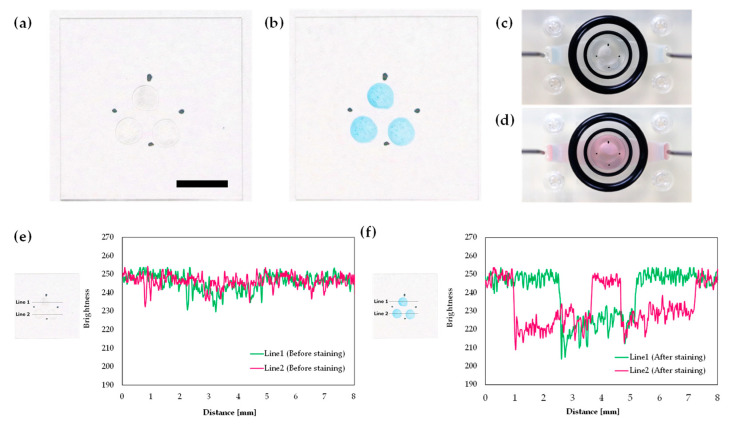
Protein spots on a cover glass: (**a**) before staining; (**b**) after staining by Coomassie blue dye. Topside view of the observation area of the 3D-printed cartridge with a protein spotted cover glass: (**c**) before Coomassie blue dye perfusion; (**d**) during perfusion. Brightness values of protein spots: (**e**) before staining; (**f**) after staining by Coomassie blue dye. Scale bar: 5 mm.

Protein spots on the 18 × 18 mm^2^ cover glass before and after staining by Coomassie blue dye are shown in Figure 7a,b. These spots were brightly stained blue without distortion. Images of the top side of the 3D-printed cartridge before and after perfusing Coomassie blue dye are shown in Figure 7c,d. There were no air bubbles in the observation area of the 3D-printed cartridge. We then analyzed the brightness of the protein spots using the ImageJ plot profile command with a one-pixel profile line width (Figure 7e,f). Although brightness reduction was not observed before staining, single brightness reduction with approximately 2 mm width in Line 1 and double brightness reduction with approximately 2 mm width in Line 2 were observed after staining. After using Coomassie blue dye, the 3D-printed cartridge could be washed by a commercial neutral detergent; thus, the cartridge serves as a reusable chemical reactor. These results indicate that protein spots were certainly stained by Coomassie blue dye and that the 3D-printed cartridge is applicable not only for cellular response measurements but also chemical reactions, also while preventing air-bubble invasion.

## 4. Conclusions

In this study, we successfully developed a 3D-printed bubble-free perfusion cartridge system for cell-based assays such as odorant sensing without any additional devices or chemical reactors. The cartridge prevented continuous air bubbles from entering the observation area and its bubble removal capability was independent of bubble volume. Calcium imaging of Or13a cells confirmed that the detection limit, response time, and recovery time of the designed cartridge were comparable to those of a commercial open bath chamber. In addition, we conducted calcium imaging of Or13a using the 3D-printed cartridge using a low-magnification objective lens. This measurement method could be applied to detect odorant responses of arrays of multiple cell lines. The results of protein staining by perfusing blue dye demonstrated that the cartridge can be used as a chemical and biological reactor. Therefore, the cartridge system in this study is suitable for bubble-free cellular response measurements and chemical reaction experiments. The 3D-printed cartridge system has great potential for facilitating a wide range of cell-based assays and for developing portable biosensor systems for environmental monitoring, food and water hygiene control, and toxicology testing.

## Figures and Tables

**Table 1:** Detailed bubble volume information and bubble removal results.

Bubble Volume [µL]	Number of Bubbles	Removing Results
**0.39–0.79**	**Over 100**	**Removed**
**25**	**30**	**Removed**
**250**	**5**	**Removed**

## Supplementary Materials

The following are available online at https://www.mdpi.com/1424-8220/20/20/5779/s1, Supporting figures, Video 1: Bubble removal mechanism of the 3D-printed cartridge; Video 2: Bubble removal sequence of the 3D-printed cartridge
