# Supplementary material for: 3D-Printed Bubble-Free Perfusion Cartridge System for Live-Cell Imaging
Source: Sensors (Basel). 2020 Oct 12;20(20):5779. doi: 10.3390/s20205779 (PMC7650622; doi:10.3390/s20205779)

**Supporting figures**

**3D-printed bubble-free perfusion cartridge system for live-cell imaging**

**Daigo Terutsuki** ***, Hidefumi Mitsuno and Ryohei Kanzaki** *

Research Center for Advanced Science and Technology, The University of Tokyo, 4-6-1 Komaba, Meguro-ku, Tokyo, 153-8904, Japan

*Correspondence: terutsuki@g.brain.imi.i.u-tokyo.ac.jp (D.T.); kanzaki@rcast.u-tokyo.ac.jp (R.K.)

**Fig. S1** Cross-sectional views of the 3D-printed cartridge. (a) Top view of the top layer of the 3D-printed cartridge. (b) Cross-sectional view in the A-A direction of (a). (c) Cross-sectional view in the B-B direction of (a). (d) Cross-sectional view in the vertical direction of the top layer of the 3D-printed cartridge including inverted taper flow channel structures.

**Fig. S2** (a) Cartridge setup for assay buffer perfusion with the metal tubes connected to silicone tubes and the 3D-printed fixtures. (b) The details of the 3D-printed fixture. A screw fixes the metal tube and a magnet enhances the stability of the 3D-printed fixture.

**Fig. S3** Enlarged original fluorescence images of the pseudo-colour heat maps for: (a) the 3D-printed cartridge with a 20× water-immersion objective lens over 1024 s, and (b) a 20× objective lens over 1030 s within the yellow dotted lines shown in Figure 5(a). Brightness values of the cells in the enlarged original fluorescence images measured (c) by a 20× water-immersion objective lens, and (d) by a 20× objective lens. All scale bars: 20 µm.


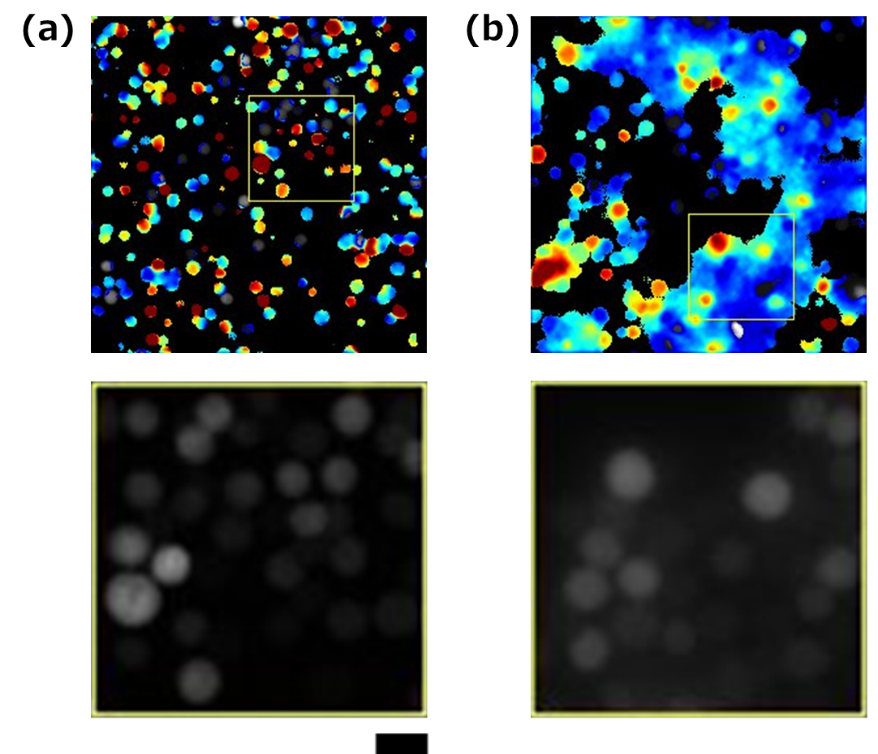

Supplement: Supplementary file 1 [file sensors-20-05779-s001.zip › Supplementary_materials_201012.docx]
